# Low maternal care enhances the skin barrier resistance of offspring in mice

**DOI:** 10.1371/journal.pone.0219674

**Published:** 2019-07-11

**Authors:** Takashi Sakamoto, Yukino Ishio, Yuiko Ishida, Kazutaka Mogi, Takefumi Kikusui

**Affiliations:** Department of Animal Science and Biotechnology, Azabu University, Sagamihara, Kanagawa, Japan; Duke University, UNITED STATES

## Abstract

Deprivation of maternal care via lack of somatosensory input causes offspring to experience adverse consequences, especially in the central nervous system. However, little is known about the developmental effect of maternal care on peripheral tissues such as the skin, which includes cutaneous sensory neurons. In the present study, we examined the involvement of maternal care in the development of the skin. We investigated offspring reared by early-weaned mother mice who spontaneously showed lower frequency of licking/grooming on nursing. Offspring of early-weaned mothers showed higher resistance against skin barrier disruption than did offspring of normally-weaned mothers, and had normal skin barrier function in the intact trunk skin. In the dorsal root ganglion of early-weaned mother offspring, we also found up-regulation of mRNA levels of the Mas-related G-protein coupled receptor B4 (MrgprB4), which is a marker of sensory neurons that detect gentle stroking. We further found that levels of MrgprB4 mRNA were correlated with the enhancement of skin resistance. The present findings suggest that maternal somatosensory inputs have a developmental impact on the cutaneous sensory neurons of the skin in offspring. Interestingly, the present results suggest that lower maternal care has a benefit on the skin resistance. This provides important information for understanding the development of peripheral tissues in offspring reared under severe conditions such as lower maternal care in the wild.

## Introduction

Maternal care is essential for appropriate development in the early stages of life. Adverse childhood experiences including neglect and abuse have been found to reduce cognitive performance, impair social development, and increase prevalence of personality disorder [1, 2]. In support of these clinical findings, animal studies have shown that daily maternal separation throughout the pre-weaning period induces dysfunction of the central nervous system (CNS) [3–9]. Additionally, deprivation of maternal care induces multiple behavioral changes in offspring [10–15]. Early-weaned female mice show lower frequency of licking/grooming (LG) when nursing their pups than did normally-weaned female mice, although time spent attending to pups and mother-off pup times are not changed [10]. Thus, the deprivation of maternal care has been shown to induce significant negative consequences on development.

Mothers provide infants with various social cues through the somatosensory, gustatory, olfactory, auditory, and visual systems. In particular, somatosensory inputs are an important component of maternal care for development [16, 17]. For example, maternal touch in children supports neurodevelopmental outcomes [18]. In rodents, maternal LG-like stimulation completely or partially restores the CNS dysfunction caused by maternal separation [3, 19–21]. It has been shown that gentle touch like LG stimulation is mainly perceived by low-threshold mechanoreceptors of the cutaneous sensory neurons expressing MAS-related G-protein coupled receptor B4 (MrgprB4) or tyrosine hydroxylase (TH) in the dorsal root ganglions (DRGs) in mice [22, 23]. It has also been reported that DRG neurons projecting to the skin significantly influence skin homeostasis [24–27]. However, little is known about the developmental effects of somatosensory inputs in the pre-weaning period on peripheral tissues, such as the skin tissue which includes cutaneous sensory neurons.

In this study, to examine the involvement of maternal care in the development of the peripheral tissue of offspring, we utilized offspring reared by early-weaned mother mice that spontaneously show lower LG when nursing [10]. A comparison between offspring of normally-weaned mother mice and offspring of early-weaned mother mice, which themselves were normally weaned, is a good model to examine the effect of maternal LG in the development. We firstly examined the maternal effect on skin barrier function in the intact skin and barrier-distracted skin, together with gene expression in the skin and the DRGs.

## Materials and methods

### Animal preparation and procedures

C57BL/6 mice obtained from Japan Clea Co., Ltd. (Yokohama, Japan) were used for all experiments. Male and female mice were pair-housed in cages (175 × 245 × 125 mm) for breeding, and pups were reared by both parents until weaning. Food and water were supplied ad libitum, and the environment was maintained at a constant temperature (24 ± 1°C) and humidity (50 ± 5%) under a 12 h light–dark cycle (lights on at 08:00). All animal experiments were approved by the Ethical Committee of Azabu University (#070412).

The procedure to obtain early-weaned mice (F1) was similar to our previous study [28]. Briefly, when female mice became pregnant, they were checked daily in the morning until parturition. For each litter, the date of birth was designated postnatal day 0 (PD0). On PD16, half of the litter was separated from each dam and assigned to the early-weaned group. The remaining pups were assigned to the normally-weaned group, cared for with standard procedures, and weaned on PD28. The early-weaned mice were fed powdered pellets until PD28. Thereafter, all pups were fed pellets. After weaning, 2–3 pups were housed together with siblings, according to sex. When both the early-weaned and normally-weaned F1 female mice were 8 weeks of age, each female was paired with a normally-weaned male mouse for breeding. All of the resulting litters (F2) were normally weaned on PD28 and housed as described above. In the present study, F2 males from both early-weaned and normally-weaned F1 mothers (EW-F2 and NW-F2) were tested in adulthood as described below.

To examine the effect of low maternal care on the development of skin tissue, we performed the following experiments using NW- and EW-F2 offspring. Adult NW- and EW-F2 offspring (34–39-week-old, NW-F2 offspring: n = 9; EW-F2-offspring: n = 7) were used in Experiment I. Trans epidermal water loss (TEWL) in the intact trunk skin was measured to evaluate skin barrier function. Mice were then killed by cervical dislocation, and the trunk skin and DRGs were harvested for histological and gene expression assessments. In Experiment II, adult NW- and EW-F2 offspring (9–11-week-old, NW-F2 offspring: n = 8; EW-F2 offspring: n = 10) were used for analysis of the developmental effect of low maternal care on the skin barrier disruption response. TEWL was measured before and after acetone-diethyl-ether (AE) treatment for 4 days on the trunk skin. Mice were then killed by cervical dislocation, and the trunk skin and DRGs were harvested for histological and gene expression assessments.

### Measurement of TEWL on the trunk skin

On the day before TEWL measurement and AE treatment, mice were anesthetized with 2% isoflurane (Wako pure chemical industries, Ltd., Osaka, Japan) and the hair on the trunk skin was shaved using electric razor. TEWL was measured using a Tewameter TM210 (Courage & Khazawa) under 2% isoflurane anesthesia. TEWL was assessed six times in each individual, and average value of the six measurements was used for analysis. In Experiment II, TEWL was measured before, and on the day after AE treatment for 4 days. The percent increase in TEWL was calculated by dividing the TEWL value after AE treatment by that before treatment in the same individual.

### AE treatment for cutaneous barrier disruption

A cotton pad soaked with a mixture of acetone and diethyl-ether (1:1) was placed on the shaved skin for 15 s under 2% isoflurane anesthesia. The treatment was performed twice daily (AM and PM) for 4 days. Although each mouse was anesthetized 8 times, it has been reported that the anesthesia did not induce significant differences in the skin microcirculation which plays a significant role in the skin homeostasis [29]. The minimum interval between AM and PM was 6 hours.

### Skin tissue histology

Skin tissue was fixed in 3.7% formalin in phosphate buffered saline (PBS) overnight at 4°C. Formalin-fixed skin tissue was cut and stained with hematoxylin and eosin (HE) at the Biopathology Institute Co., Ltd. (Oita, Japan). Photomicrographs were taken using a BZ-X710 microscope (KEYENCE, Japan) at magnification of x 400.

### Quantification of mRNA in the trunk skin and T2-L2 DRGs

Trunk skin and T2-L2 DRGs were dissected from the mice. Total RNA was isolated from trunk skin using an RNeasy Fibrous Mini Kit (QIAGEN, Venlo, Netherlands), and from DRGs using an RNeasy Mini Kit (QIAGEN). Concentration and purity were assessed using a NanoDrop-1000 (Thermo Scientific, Inc., MA, USA). Total RNA was reverse-transcribed and amplified using a High-Capacity cDNA Reverse Transcription Kit (Thermo Fisher Scientific, Inc.). The quantity of mRNA was analyzed using TaqMan gene expression assays, TaqMan Master Mix, and a 7500 Fast Real-time PCR system (Thermo Fisher Scientific, Inc.). The following TaqMan gene expression assays were used: Mm00498375_m1 (Transglutaminase 1, Tgm1), Mm00493699_m1 (Tight junction protein 1, Tjp 1), Mm00516701_m1 (Claudin-1, Cld-1), Mm00515514_s1 (Claudin-4, Cld-4), Mm01716522_m1 (Filaggrin, Flg), Mm01962650_s1 (loricrin, Lor), Mm01305291 (Keratin 5, K5), Mm00492992_g1 (Keratin 1, K1) and Mm00515219_s1 (Involucrin, Ivl) for the skin, and Mm01701887_g1 (Mas-related G-protein coupled receptor member B4, MrgprB4) and Mm00447557_m1 (Tyrosine hydroxylase, Th) for the DRGs. The quantity of mRNA was normalized to that of ribosomal protein P0 (Rplp0, Mm00725448_s1) in individual samples [30–32]. For comparison between NW- and EW-F2 offspring, data are presented as the ratio of the values to the normalized values of NW-F2 offspring without AE treatment in Experiment I. For correlation analysis, relative gene expression data were calculated by dividing by the amount of Rplop0 mRNA in individual samples.

### Statistical analysis

Results are expressed as means ± SE. All statistical analyses were performed using GraphPad Prism version 6.0 (Graphpad Software Inc., CA, USA). Two-way repeated measures and factorial ANOVA with Bonferroni and Tukey’s *post hoc* test were performed to compare multiple groups, and unpaired t-tests with two-tailed distribution were used to assess statistical significance when two group was compared. Pearson’s correlation coefficient was used to assess associations. A significance threshold of α = 0.05 was used.

## Results

### Skin barrier function, skin histology and mRNA expression in the trunk skin of adult mice reared by normally- or early-weaned mothers

**Fig 1:**
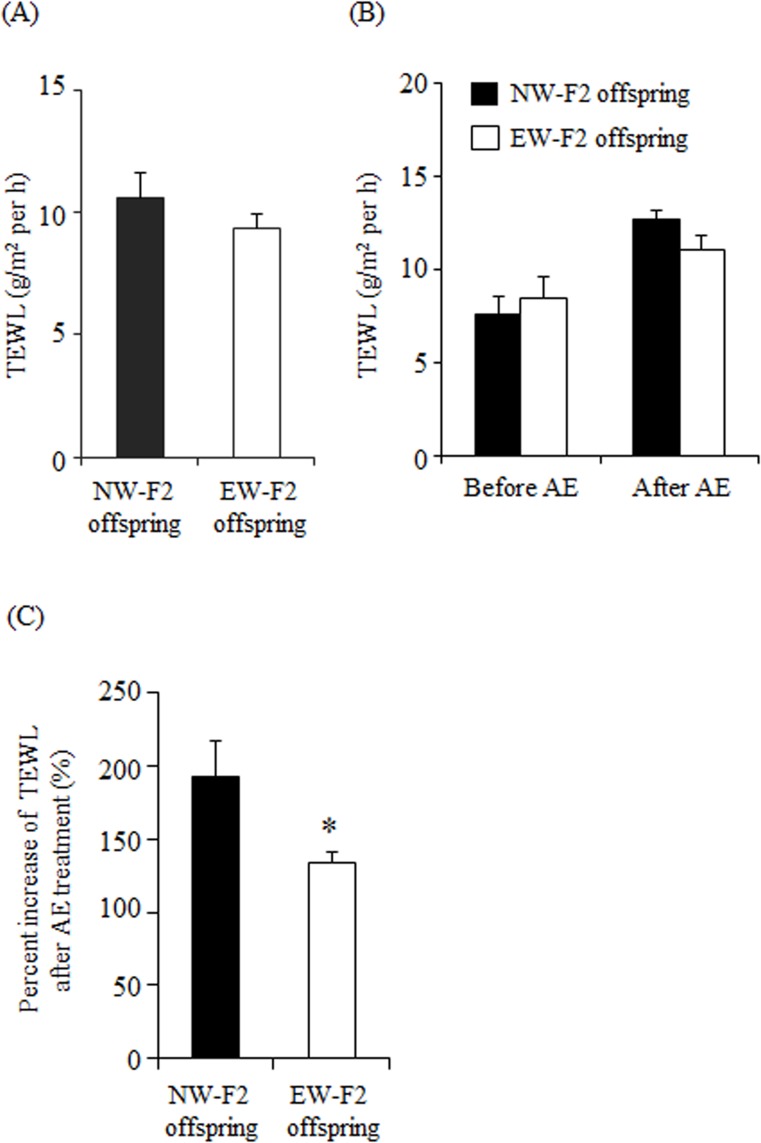
TEWL value in the intact trunk skin and percent increase of TEWL after AE treatment. (A) TEWL value in the intact trunk skin. (B) TEWL values before and after AE treatment in NW- (black) and EW-F2 (white) offspring. Two-way repeated measures ANOVA showed main effect of AE treatment. (C) Percent increase in TEWL after AE treatment. Percent increase of TEWL was calculated in the same individuals. All data are presented as the mean ± SE (n = 7–10, *: *p* < 0.05).

The integrity of barrier function was assessed by TEWL. A high TEWL value reflects reduced skin barrier function with high permeability [33]. TEWL values of the intact trunk skin were not significantly different between NW- and EW-F2 offspring (NW-F2 offspring: 10.62 ± 0.98 g/m^2^ per h; EW-F2 offspring: 9.35 ± 0.28 g/m^2^ per h, *p* = 0.28, unpaired t-test, Fig 1A), indicating that spontaneous lower LG behavior while nursing does not alter skin barrier function in the intact skin.

We then examined differences in skin resistance against AE treatment. It has been shown that AE treatment increases TEWL values [34, 35]. Consistently, two-way repeated measures ANOVA detected a significant main effect of AE treatment (F (1, 16) = 34.88, *p* < 0.0001). There was no significant effect of maternal care (F (1, 16) = 0.1557, *p* = 0.6984) or interaction between maternal care and AE treatment (F (1, 16) = 3.792, *p* = 0.070, NW-F2 offspring: 7.6 ± 0.98 g/m^2^ per h [before AE], 12.7 ± 1.1 g/m^2^ per h [after AE]; EW-F2 offspring: 8.46 ± 0.48 g/m^2^ per h [before AE], 11.0 ± 0.80 g/m^2^ per h [after AE], Fig 1B). On the other hand, the percent increase of TEWL by AE treatment in EW-F2 offspring (133.2 ± 7.2%) was significantly lower than that of NW-F2 offspring (191.7 ± 24.4%, *p* < 0.05, unpaired t-test, Fig 1C).

**Fig 2:**
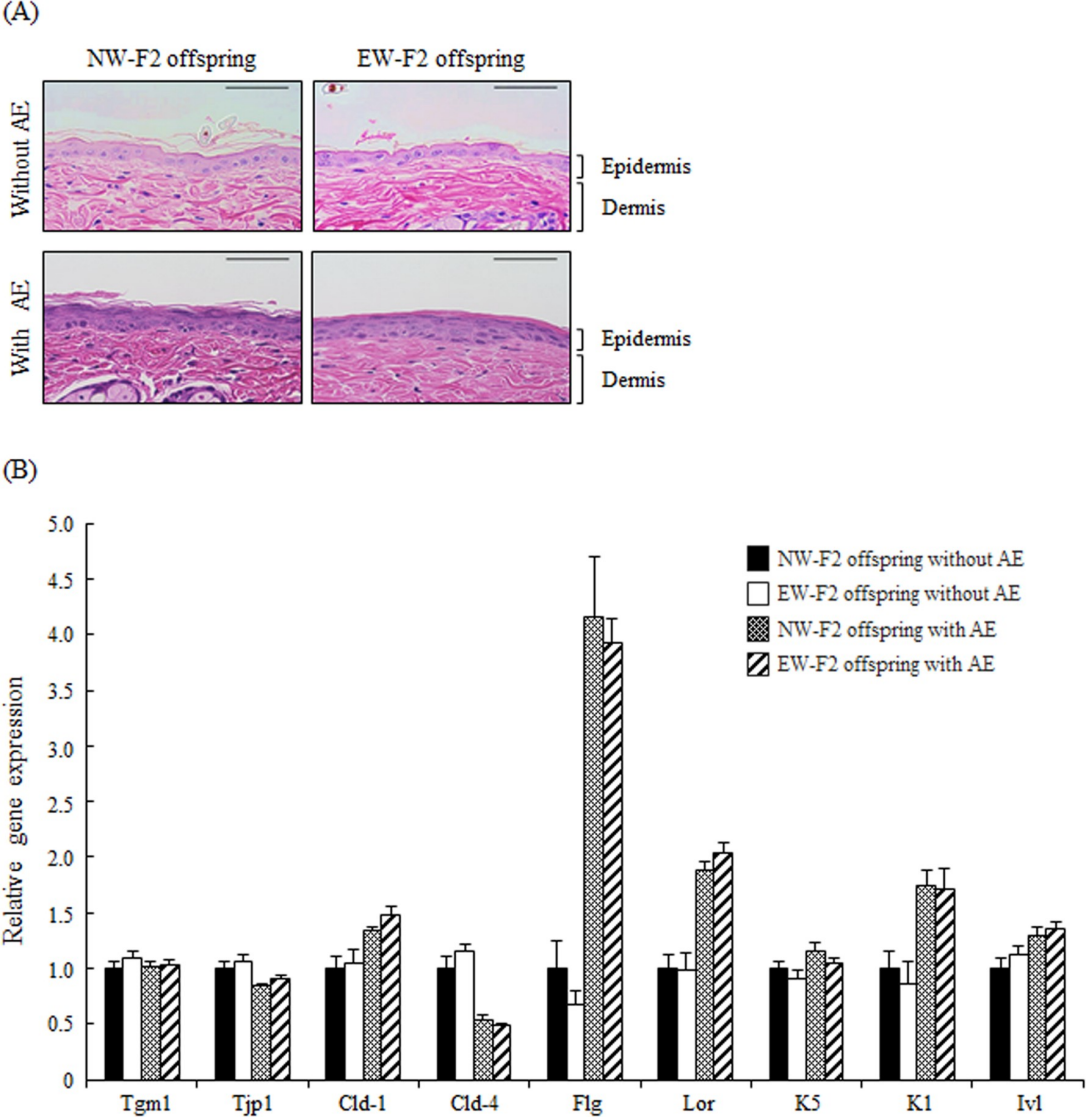
Skin histology and expression of mRNA in the trunk skin without and with AE treatment. (A) Representative HE staining of the trunk skin with and without AE treatment in NW- and EW-F2 offspring (bar indicating 50 μm). (B) Expression of mRNA in the trunk skin with and without AE treatment in NW- and EW-F2 offspring. Gene expression data are presented as a ratio of the amount of mRNA in NW-F2 offspring before AE treatment. Two-way factorial ANOVA showed significant main effect of AE treatment in K5, K1, Ivl, Tjp1, Cld-1, Cld-4, Flg and Lor. All data are presented as the mean ± SE (n = 7–10).

We performed hematoxylin-eosin (HE) staining in intact trunk skin in Experiment I and AE treated trunk skin in Experiment II. Representative data is shown in Fig 2A. Epidermal hyperplasia was observed in the spinous-granular layers in the AE treated skin of both NW- and EW-F2 offspring, and the degree of hyperplasia were not different between groups. In addition to histological analysis, we quantified mRNA levels related to differential markers of skin keratinocytes and skin barrier function (S1 Table). Keratin-5 (K5) is a differential maker of basal layer, and keratin-1 (K1) and involucrin (Ivl) are differential markers of spinous-granular layers of the epidermis [36]. Tight junction protein 1 (Tjp1), and Claudin-1 and -4 (Cld-1 and -4) are components of tight junctions which are complex cell-cell junction and form a barrier in the glanular layer of the skin [37, 38]. Transglutaminase-1 (Tgm1), Filaggrin (Flg) and Loricrin (Lor) are expressed in the spinous-granular layers [39, 40]. Tgm1 is involved in the formation of the cornified cell envelope which is a critical structure to barrier function in the skin epidermis [38]. Flg and Lor are terminally differentiating structural proteins contributing to the protective barrier function of the skin [40, 41]. Two-way factorial ANOVA tests detected a significant main effect of AE treatment in K5, K1, Ivl, Tjp1, Cld-1, Cld-4, Flg and Lor mRNA levels, but not in Tgm1 mRNA levels (S2 Table). There were no significant main effect of maternal care or interaction between maternal care and AE treatment in the mRNA levels of all genes examined.

### Expression of mRNA in T2-L2 DRGs in adult mice reared by normally- or early-weaned mother mice

**Fig 3:**
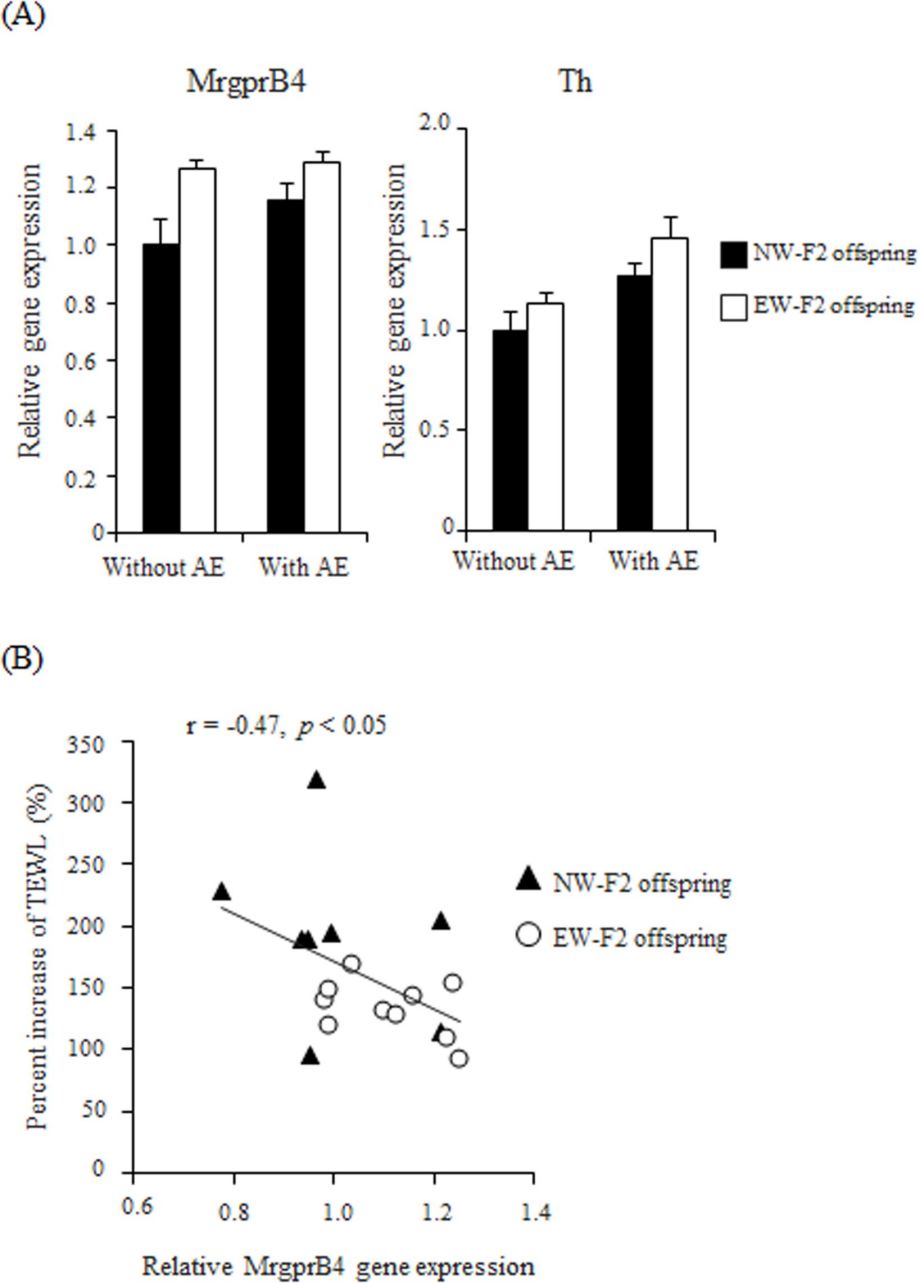
Expression of MrgprB4 and Th mRNA in offspring, and correlation between MrgprB4 mRNA and percent increase in TEWL after AE treatment. (A) Expression of MrgprB4 and Th mRNA in T2-L2 DRGs in NW- (black) and EW- (white) F2 offspring with and without AE treatment. Gene expression data are presented as a ratio of the amount of mRNA in NW-F2 offspring without AE treatment. Two-way factorial ANOVA showed significant main effect of maternal care in MrgprB4, and significant main effect of AE treatment in Th. Data are presented as the mean ± SE (n = 7–10). (B) Correlation between the relative amounts of MrgprB4 mRNA in the T2-L2 DRGs from AE treated mice and the percent increase of TEWL after the treatment.

We quantified mRNA levels of low-threshold mechanoreceptors, MrgprB4 and Th, in DRGs in AE untreated (Experiment I) and treated offspring (Experiment II). In MrgprB4 mRNA levels, two-way factorial AVOVA detected a significant main effect of maternal care (F (1, 30) = 9.404, *p* = 0.0046), and there was no significant AE treatment effect (F (1, 30) = 1.974, *p* = 0.1703) or interaction between maternal care and AE treatment (F (1, 30) = 1.165, *p* = 0.289, NW-F2 offspring: 1.00 ± 0.09 [without AE], 1.15 ± 0.06 [with AE], EW-F2 offspring: 1.26 ± 0.03 [without AE], 1.28 ± 0.04 [with AE], Fig 3A). On the other hand, with respect to Th mRNA levels, two-way factorial ANOVA detected a significant main effect of AE treatment (F (1, 30) = 12.23, *p* = 0.0015), and there was no significant effect of maternal care (F (1, 30) = 3.451, *p* = 0.073) or interaction (F (1, 30) = 0.1428, *p* = 0.7082, NW-F2 offspring: 1.00 ± 0.08 [without AE], 1.27 ± 0.07 [with AE], EW-F2 offspring: 1.13 ± 0.05 [without AE], 1.46 ± 0.10 [with AE], Fig 3A). Interestingly, further analysis revealed that the amount of MrgprB4 mRNA in offspring with AE treatment showed significant negative correlation with the percent increase in TEWL by AE treatment (*r* = -0.47, *p* < 0.05, Fig 3B). Nonetheless, there was no significant correlation between the amount of MrgprB4 mRNA in the offspring without AE treatment and the TEWL value in the intact trunk skin (*r* = -0.43, *p* = 0.09). In terms of Th mRNA levels, there was no significant correlation between mRNA levels in the offspring without AE treatment and TEWL values in the intact skin (*r* = -0.32, *p* = 0.22), or between mRNA levels in the offspring with the treatment and the percent increase of TEWL (*r* = -0.19, *p* = 0.46).

## Discussion

We investigated the effects of maternal care in the development of peripheral tissues by analyzing offspring of early-weaned mother mice, which show low LG on nursing. We found that these offspring showed normal skin barrier function in the intact trunk skin, and higher resistance against the AE treatment than offspring from normally-weaned mothers. Furthermore, offspring of early-weaned mothers had higher mRNA levels of MrgprB4 in the DRGs. Additionally, the levels of MrgprB4 mRNA expression in the DRGs were correlated with skin resistance. Although some reports has suggested that epigenomic changes in parents are transmitted to offspring [42, 43], such the trans-generational epigenomic inheritance is still controversial. Our findings suggests that maternal care has a developmental effect on peripheral tissues of offspring, and low maternal care leads to improved skin resistance under severe conditions.

In the present study, we found that the percent increase in TEWL was lower in EW-F2 offspring after AE treatment, although TEWL values in the intact skin were not different between both maternal care groups. This clearly suggests that skin resistance against AE treatment was enhanced in offspring of early-weaned mother mice, although skin barrier function in the intact skin was not affected. It has been previously reported that LG stimulation during the pre-weaning period has effects on the development of the CNS [5–11]. To the best of our knowledge, this is the first report suggesting that LG stimuli also influence the development of skin function.

Similarly, the histological characteristics and mRNA levels known as makers for epidermal differentiation and skin barrier functions were examined in the offspring of normally- and early-weaned mothers. With AE treatment, epidermal hyperplasia was observed in the spinous-granular layers. Consistently, two-way ANOVA showed significant main effect of AE treatment in almost gene examined, but there was no significant maternal care effect or interaction between AE treatment and maternal care in those genes. That is, mRNA levels of the epidermal differential markers in the skin (K5, K1 and ivl) and epidermal barrier structural proteins expressed in the spinous-granular layers (Flg and Lor) were higher in the AE-treated skin. Moreover, in the tight junction protein, Cld-1 mRNA levels were higher in the AE treated skin. On the other hand, Cld-4 and Tjp1 mRNA levels were lower in the AE treated skin. Tgm1 mRNA levels were not changed by AE treatment. These suggest that AE treatment caused structural and functional changes. On the other hand, the results of two-way ANOVA also suggested that enhancement of skin resistance in the offspring of early-weaned mothers was not depending on those changes of gene expression in the skin.

Interestingly, we found the significant main effect of maternal care in mRNA levels of MrgprB4 in DRGs by two-way ANOVA analysis, but there was no significant main effect of AE treatment or interaction between maternal care and AE treatment. That is, mRNA levels of MrgprB4 in DRGs were higher in offspring of early-weaned mothers. These suggest that mRNA levels of MrgprB4 was negatively regulated by LG stimuli in pre-weaning period. It has been previously reported that maternal separation up-regulates the gene expression of voltage-gated channel in colonic DRGs in rat offspring, implying that maternal care down-regulates that gene expression in DRGs [44]. These suggest that LG stimulation could down-regulate the expression of certain genes within DRGs. In the present study, it was further found that MrgprB4 mRNA levels were significantly correlated with the percent increase in TEWL after AE treatment, but not with TEWL values in the intact skin. This suggests that MrgprB4 mRNA levels in DRGs were not related to skin barrier function in normal conditions but were correlated with skin resistance against AE treatment. The selective and artificial activation of MrgprB4+ neurons can result in the promotion of conditioned place preference, suggesting that MrgprB4-expressing sensory neurons participate in positive affective valence [24], but little is known about the involvement of these neurons in skin resistance. The present results suggest that the activation of MrgprB4+ sensory neurons might contribute to the enhancement of skin resistance. It has been shown that activation of nociceptive neurons in the skin induces the release of inflammatory neuropeptides such as substance P, thereby causing neurogenic skin inflammation and reduction of skin barrier function [26, 27]. These studies provide support for the notion that the primary sensory neurons influence the cutaneous responses. Further investigation into the direct effect of MrgprB4+ neuronal activation on skin resistance is needed. Our studies suggest that the action of MrgprB4+ sensory neurons provide benefits to physical function as well as cognitive function.

Notably, low maternal care enhanced the ability of skin resistance in the present study. This may have physiological significance from an ecological point of view. It has been reported that all organisms show a remarkable flexibility that allows them to survive under severe conditions such as an insufficient maternal care in the wild environment [45–47]. Although previous studies demonstrated that early-weaned rodents show increased anxiety-like behaviors [48, 49], this change could be explained by their potential ability to elevate vigilance against severe environmental conditions such as their mother had to wean their pups earlier. That is, this notion suggest that lower maternal care could increase behavioral flexibility of offspring. In the present study, higher mRNA level of MrgprB4 in offspring of early-weaned mothers might be attributed to increased flexibility to adapt to lower somatosensory input. Our results may have important implications for understanding the development of peripheral function in offspring under insufficient maternal care.

In summary, we revealed that low maternal care enhances skin barrier resistance, and was correlated with higher levels of MrgprB4 mRNA. This study provides new insight into the developmental benefit of low maternal care during the pre-weaning period on skin tissue, including cutaneous sensory neurons.

## Supporting information

S1 Table Relative gene expression levels in the skin without and with AE treatment. (DOCX)

S2 Table Summary of two-way ANOVA analysis of gene expression levels in the skin. (DOCX)
