# Supplementary material for: Low maternal care enhances the skin barrier resistance of offspring in mice
Source: PLoS One. 2019 Jul 11;14(7):e0219674. doi: 10.1371/journal.pone.0219674 (PMC6624014; doi:10.1371/journal.pone.0219674)
Supplement: S1 Table — (DOCX) [file pone.0219674.s001.docx]

**S1 Table.**

**Relative gene expression levels in the skin without and with AE treatment.**

Without AE treatment With AE treatment

NW-F2 offspring EW-F2 offspring NW-F2 offspring EW-F2 offspring

(n = 9) (n = 7) (n = 8) (n = 10)

K5 1.00 ± 0.07 0.91 ± 0.08 1.15 ± 0.07 1.05 ± 0.05

K1 1.00 ± 0.15 0.86 ± 0.19 1.75 ± 0.14 1.71 ± 0.18

Ivl 1.00 ± 0.09 1.13 ± 0.08 1.29 ± 0.07 1.35 ± 0.06

Tjp1 1.00 ± 0.07 1.07 ± 0.06 0.84 ± 0.02 0.91 ± 0.03

Cld1 1.00 ± 0.11 1.05 ± 0.12 1.34 ± 0.03 1.48 ± 0.08

Cld4 1.00 ± 0.11 1.16 ± 0.06 0.54 ± 0.04 0.49 ± 0.01

Tgm1 1.00 ± 0.06 1.09 ± 0.06 1.01 ± 0.05 1.03 ± 0.05

Flg 1.00 ± 0.25 0.67 ± 0.13 4.16 ± 0.54 3.93 ± 0.21

Lor 1.00 ± 0.12 0.98 ± 0.16 1.88 ± 0.08 2.04 ± 0.10

All data are presented as the mean ± SE.
