# Supplementary material for: Low maternal care enhances the skin barrier resistance of offspring in mice
Source: PLoS One. 2019 Jul 11;14(7):e0219674. doi: 10.1371/journal.pone.0219674 (PMC6624014; doi:10.1371/journal.pone.0219674)
Supplement: S2 Table — (DOCX) [file pone.0219674.s002.docx]

**S2 Table.**

**Summary of two-way ANOVA analysis of gene expression levels in the skin.**

Gene Source of variance F df p

K5 Maternal care 2.105 1, 30 0.1572

AE treatment 4.760 1, 30 0.0371

Maternal care x AE treatment 0.009483 1, 30 0.9230

K1 Maternal care 0.2612 1, 30 0.6130

AE treatment 21.90 1, 30 < 0.0001

Maternal care x AE treatment 0.08143 1, 30 0.7773

Ivl Maternal care 1.547 1, 30 0.2232

AE treatment 11.31 1, 30 0.0021

Maternal care x AE treatment 0.1921 1, 30 0.6644

Tjp1 Maternal care 1.087 1, 30 0.3056

AE treatment 8.179 1, 30 0.0076

Maternal care x AE treatment 0.08619 1, 30 0.7711

Cld1 Maternal care 1.130 1, 30 0.2963

AE treatment 18.34 1, 30 0.0002

Maternal care x AE treatment 0.2547 1, 30 0.6175

Cld4 Maternal care 0.6618 1, 30 0.4223

AE treatment 67.59 1, 30 < 0.0001

Maternal care x AE treatment 2.292 1, 30 0.1405

Tgm1 Maternal care 0.8395 1, 30 0.3669

AE treatment 0.2082 1, 30 0.6515

Maternal care x AE treatment 0.5038 1, 30 0.4833

Flg Maternal care 0.7710 1, 30 0.3869

AE treatment 101.3 1, 30 < 0.0001

Maternal care x AE treatment 0.02277 1, 30 0.8811

Lor Maternal care 0.3933 1, 30 0.5353

AE treatment 70.49 1, 30 < 0.0001

Maternal care x AE treatment 0.5985 1, 30 0.4452
